# Community-Integrated Intermediary Care (CIIC) Service Model to Enhance Family-Based, Long-Term Care for Older People: Protocol for a Cluster Randomized Controlled Trial in Thailand

**DOI:** 10.2196/20196

**Published:** 2021-03-24

**Authors:** Myo Nyein Aung, Saiyud Moolphate, Motoyuki Yuasa, Thin Nyein Nyein Aung, Yuka Koyanagi, Siripen Supakankunti, Ishtiaq Ahmad, Ryoma Kayano, Paul Ong

**Affiliations:** 1 Advanced Research Institute for Health Sciences and Faculty of International Liberal Arts Juntendo University Tokyo Japan; 2 Department of Public Health Faculty of Science and Technology Chiang Mai Rajabhat University Chiang Mai Thailand; 3 Faculty of International Liberal Arts Juntendo University Tokyo Japan; 4 Department of Public Health Juntendo University Tokyo Japan; 5 Tokyo Ariake University of Medical and Health Sciences Tokyo Japan; 6 Faculty of Economics Chulalongkorn University Bangkok Thailand; 7 Centre for Health Development World Health Organisation Kobe Japan

**Keywords:** aging, Asia, care prevention, health promotion, long-term care, implementation research

## Abstract

**Background:**

Thailand is one of the most rapidly aging countries in Asia. Traditional family-based care, which has been the basis of most care for older people, is becoming unsustainable as families become smaller. In addition, women tend to be adversely affected as they still form the bulk of caregivers for older people, and many are likely to exit the labor market in order to provide care. Many family caregivers also have no or minimal training, and they may be called upon to provide quite complex care, increasing the proportion of older people receiving suboptimal care if they rely only on informal care that is provided by families and friends. Facing the increasing burden of noncommunicable diseases and age-related morbidity, Thai communities are increasingly in need of community-integrated care models for older persons that can link existing health systems and reduce the burden upon caring families. This need is common to many countries in the Association of Southeast Asian Nations (ASEAN).

**Objective:**

In this study, we aimed to assess the effectiveness of a community-integrated intermediary care (CIIC) model to enhance family-based care for older people.

**Methods:**

This paper describes a cluster randomized controlled trial comprised of 6 intervention clusters and 6 control clusters that aim to recruit 2000 participants in each arm. This research protocol has been approved by the World Health Organization Ethics Review Committee. The intervention clusters will receive an integrated model of care structured around (1) a community respite service, (2) the strengthening of family care capacity, and (3) an exercise program that aims to prevent entry into long-term care for older people. Control group clusters receive usual care (ie, the current system of long-term care common to all provinces in Thailand), consisting principally of a volunteer-assisted home care service. The trial will be conducted over a period of 2 years. The primary outcome is family caregiver burden measured at a 6-month follow-up, as measured by the Caregiver Burden Inventory. Secondary outcomes consist of biopsychosocial indicators including functional ability, as measured using an activity of daily living scale; depression, as measured by the Geriatric Depression Scale; and quality of life of older people, as measured by the EuroQol 5-dimensions 5-levels scale. Intention-to-treat analysis will be followed.

**Results:**

The CIIC facility has been established. Community care prevention programs have been launched at the intervention clusters. Family caregivers are receiving training and assistance. However, the COVID-19 pandemic delayed the intervention.

**Conclusions:**

Since ASEAN and many Asian countries share similar traditional family-based, long-term care systems, the proposed CIIC model and the protocol for its implementation and evaluation may benefit other countries wishing to adopt similar community-integrated care models for older people at risk of needing long-term care.

**Trial Registration:**

Thai Clinical Trials Registry TCTR20190412004; http://www.thaiclinicaltrials.org/#

**International Registered Report Identifier (IRRID):**

DERR1-10.2196/20196

## Introduction

### Background

Thailand’s population is estimated to be the third most rapidly aging in the world [1]. While it has a strong health system with a well-established system of universal health coverage, Thailand still needs to develop a long-term care (LTC) system and LTC insurance program. Policies are also needed to sustainably finance an effective LTC model for an estimated 11 million older people in 2020 [2]. Family-based, long-term care (FLTC) is the current model for the majority of LTC in Thailand.

Families form the backbone of the LTC system, a trait that is common to many Southeast Asian countries [3]. However, as families become smaller in Thailand, the sustainability of FLTC is challenged by human resources and its suitability as the technical needs for the care of older people become more complex. Vastly differing levels of education and care competencies found within family caregivers may cause inequalities in the care of older people across lower-resource and lower-income countries [4]. Furthermore, increasing caregivers’ burden and opportunity cost pose a key challenge to fast-aging countries where informal care in FLTC is the backbone of aging care. Such burden leads to job loss of the caregivers, abuse of older persons, and caregiver burnout. Interventions promoting the care capacity of informal care in FLTC become essential. Moreover, community-integrated respite service is required to reduce caregivers’ burden, sustain FLTC, and promote both older persons’ and caregivers’ health.

Among the Asian countries except Japan, FLTC is the common choice for most older adults. In some cases, it may be the only choice [3,4]. Recent literature reports studies of LTC models that are well established in high-income countries and compared them to the system of informal family caregiving in many Asian countries such as Thailand [4,5]. Aging and LTC in low-income Asian countries are insufficient for institution-based care and facilities, with policies tilting more towards relying on families and unpaid caregivers. However, many studies reported that FLTC in Asia had a greater positive impact on the psychosocial well-being of older adults [4,6].

In Thailand, as in many Association of Southeast Asian Nations (ASEAN) countries, families, particularly adult children, play a major role in giving care to older adults [3]. There are challenges to FLTC in the context of the Thai society. Adult children may migrate to work in geographically distant places, and concurrently, the sizes of families are in decline [7,8]. Moreover, the FLTC model prevents women and carers from entering the labor force and can place significant unmitigated burden and stress on those providing care. Based on predictions of increasing burden on family caregivers [6,7] it is important to strengthen the traditional FLTC model by initiating a feasible, formal LTC service and care prevention program.

If the weakness in FLTC in terms of technical and professional needs can be strengthened through community-based innovation, the results could be reduced burden of family caregivers, a positive impact on the biopsychosocial outcomes of older adults, and potentially improved healthy aging in communities. Therefore, research assessing the efficacy of innovative LTC models that are contextually relevant is urgently required to assist policymakers who will need to create evidence-based policies to strengthen or build LTC systems including FLTC.

In the context of limited facilities for LTC amidst a growing population of older people, intermediary care models can be an attractive option [9]. A recent study in Australia reported the promising impact of intermediary care centers on reducing family burden and improving the well-being of older adults [10]. This study suggested that intermediary care service with early detection and preventive services may be cost-effective.

Whether the presence of a community-integrated intermediary care center in a district can reduce the burden of family caregivers and promote the functional ability and quality of life (QOL) of older adults in the communities of Thailand is an interesting research question that has not yet been researched systematically. This study aims to assess the efficacy of a community-integrated intermediary care (CIIC) model and compare it to the existing traditional family care model in Thailand.

**Figure 1:**
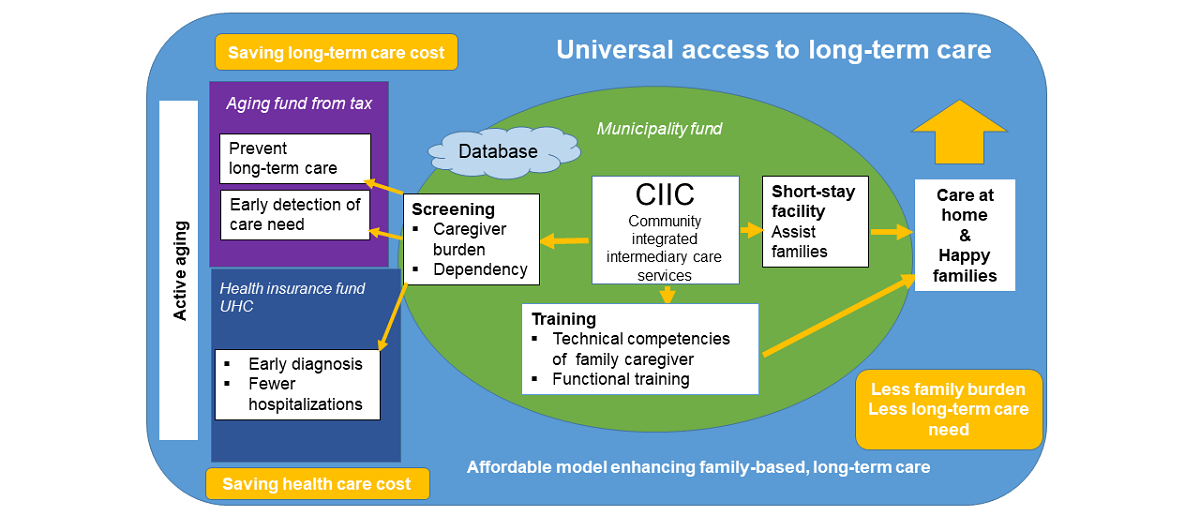
Conceptual framework of the Community-Integrated Intermediary Care (CIIC) model for older persons. UHC: universal health coverage scheme.

The CIIC model (Figure 1) is carefully designed to link the existing health care system and services in Thailand to the LTC needs of older people. The screening activity of the CIIC aims to screen for dependency and continually assess family caregiver burden, which could detect early low-level care needs and thereby prevent the future need for more intensive LTC.

In Thailand, when environmental alterations are required to accommodate the needs of an older person, such as house management, handrails, and toilet and bathing modifications, district and local municipal funds usually support these needs. If older adults need assistive devices for instrumental activities of daily living (ADL), such as wheelchairs and walking sticks, there is an insurance scheme supported by national taxation (Figure 1). The proposed CIIC service will identify those who are in need and link these older people and their families to existing funds and providers. Moreover, community-based screening activities may identify persons with hypertension, diabetes, and other medical problems. The CIIC team can refer these individuals to the primary health care unit or the community hospital where all Thai citizens can access medical care as part of universal health coverage under the national health insurance program.

Recent policy in Thailand tends to support local governments in setting up centers for aging and LTC through social security insurance. This bill was passed to support municipal governments in the provision of LTC. Additionally, local governments also have their own budgets. However, there is a gap between the FLTC system and its integration with community-based health and LTC services that are managed by local government and municipalities. CIIC is expected to provide a model filling this gap (Figure 1)**.**

### Study Hypothesis, Objectives, and Rationale

#### Hypothesis

Improved competencies of family caregivers may reduce their stress and burden and promote the well-being of older adults as well as increase caregivers’ productivity. Functional training exercises for older people will preserve active aging whereas better home care will prevent LTC needs and hospitalization and could decrease health care costs. We hypothesized that there will be differences between the intervention clusters utilizing CIIC services and control clusters in terms of family caregiver burden, functional ability of the older persons, and their QOL.

Such an intervention will be evaluated in a randomized controlled trial, but individual randomization is not realistic and threatens the validity of the results by contamination. Therefore, a clustered randomized controlled design will recruit eligible villages and consented community residents, older persons, within the village as a cluster.

#### Objectives

This study aims to assess the effectiveness of a CIIC facility and its functions to assist families providing LTC for older adults in terms of reducing caregivers’ burden as the primary outcome. Another objective is to evaluate the effectiveness of the CIIC model in terms of the following secondary outcomes: impact on ADL, depression, and QOL of older people.

## Methods

### Trial Design

This is a 2-arm, parallel-design, interventional study designed as a cluster randomized controlled trial. A total of 4000 participants will be recruited in 12 clusters, 6 in the intervention group with 2000 older adults and 6 in the control group with 2000 older adults in Chiang Mai province, Thailand.

The unit of randomization, cluster, is a village. Participants in intervention clusters will receive the CIIC intervention after screening for eligibility. The eligibility criterion for a cluster is a village that has more than 300 older persons over 60 years of age at the time of randomization.

### Inclusion Criteria for Study Participants

The inclusion criteria are >60 years of age, has a family caregiver(s), either male or female, and resident in study site districts.

### Exclusion Criteria

People with the following characteristics will be excluded: lack of informed consent by those >60 years old or their family caregivers, cannot understand the explanation for informed consent despite being provided with language support, in a household without an older person >60 years old, or cognitive impairment or severe impairment of decision-making abilities.

### Study Setting

Chiang Mai, Northern Thailand is the location of the study. There are 284,457 older adults in Chiang Mai city according to the 2019 provincial report. Older adults account for 18% of the population, which is higher than the national proportion of people ≥60 years old (16%). Chiang Mai is culturally similar to many neighboring ASEAN countries in terms of caring for older parents as a family tradition and social value.

Two subdistricts involved in the study, sub-district XXX (10 villages) for intervention and subdistrict YYY (15 villages) for control, are in the same Muang Chiang Mai district of Chiang Mai province. They are geographically distant from each other. They have similar demographic and sociocultural characteristics. Both subdistricts have a similarly sized population (>18,000 people), with similar aging rates (>18% of the population is >60 years old), according to local population statistics. People in both subdistricts are mostly Thai people who have access to health care services through the national health insurance system and social welfare services for older people covered by municipality funds.

### Intervention

The intervention consists of (1) screening and assessment of family burden and LTC capacity, (2) care capacity building for family caregivers, (3) care prevention and functional training for older adults, and (4) a respite care service for families with caregiving difficulties.

#### Care in Both Arms

In both arms, a basic health check, blood pressure check, and BMI assessment will be provided for the study participants. If any disease is suspected through the health check, appropriate referrals to existing health care services will be provided. This is considered a benefit for all study participants. The control group will receive the same assessment with an explanation that the assessment is being conducted as part of a survey and the result will be utilized for research purposes. They also receive the benefit of the additional health check followed by appropriate referrals to health care professionals. Control arm participants will receive usual care (ie, the current system of LTC common to all provinces in Thailand), consisting principally of a volunteer-assisted home care service in addition to traditional family-based home care. The differences between the 2 arms are the newly launched CIIC services, as described in the following sections.

#### Screening and Assessment of Family Burden and LTC Capacity

After obtaining informed consent, screening of the families will be conducted to assess the capacity for and burden of caregiving for each family with older adults. All older adults and caregivers in each cluster who have provided informed consent will be screened for ADL status and health-related QOL utilizing the EuroQol 5-dimensions 5-levels (EQ-5D-5L) for older adults, and caregiver burden will be measured with the Caregiver Burden Inventory (CBI). The results will be utilized as a baseline assessment from which to measure the overall impact of the CIIC center.

#### Care Capacity Building for Family Caregivers

Based on the screening, a caregiving capacity building educational program will be provided to the family caregivers of dependent older adults (Table 1), based on the results of a recent study that indicated challenges with FLTC are due to a lack of education and technical competencies of family caregivers [11].

**Table 1:** Content of the capacity building and training in the Community-Integrated Intermediary Care (CIIC) service model.

Category	Households with dependent older adults	Households with active or less dependent older adults
Whom to train	Family caregiver	Older adults
What to train	Prioritization of conditions for individually tailored care needs	Functional training to preserve functional ability and prevent the need for long-term care
How	Training delivered at home or can be group training, depending on the individual situation and specific care need	Group training 2 times a week as a care prevention exercise program
Who will train	CIIC nurse or assistant	CIIC nurse or assistant or invited experts such as a physical therapist or physical educationist

#### Care Prevention Exercise Program

Several exercise programs for preventing frailty and LTC will be provided to all the older adults in each intervention cluster (Table 2). The exercise program is based on a recent Japanese study that showed a positive, significant effect with 3 months of training on muscle strength, function, and cognitive [12,13]. A Japanese expert in this training will develop sets of exercises suitable for Thai older adults and train CIIC staff and community volunteers who will then become the trainers in the implementation of the study. Several awareness campaigns to motivate community older adults to become physically active will be conducted to increase access to the exercise program. Along with the implementation of the training, several physical functions (grip strength, flexibility, functional reach, static balance, walkability, and timed up and go tests) will be measured to assess the effect of the training.

The capability of participants to engage with the exercises will be screened before the training by a doctor or nurse. Blood pressure and heart rate will be measured, and a safety checklist will be completed as part of a safety assessment. Emergency response training or refresher courses such as for the provision of cardiopulmonary resuscitation and utilization of automated external defibrillator devices will be provided. The maximum size of one training class is 30 people. One session of training will comprise 10 minutes of stretching exercises, 30 minutes of functional training and exercises, followed by 10 minutes of cooling down and stretching. A total of 24 sessions (2 sessions per week for 12 weeks) will be provided.

The 30 minutes of functional training will entail a set of very light exercises for muscle flexibility as well as exercises to improve joint range to help maintain daily mobility and functioning. Very mild resistance training using soft rubber bands (Theraband) will be performed. It aims at maintaining and improving physical fitness especially for frailer older people and promotes personal exercise habits. This is one strategy for the prevention of the need for LTC.

Care-prevention exercises will be primarily delivered as community group exercise in each cluster in the intervention arm. In addition, participants will be offered an exercise poster, DVD, and safety instruction in order to practice the exercises at home.

In case the intervention proves to be beneficial, caregivers who could not access the study because the older person refused to participate will be informed that they can receive the training if they are still interested.

**Table 2:** Tentative plan for a Community-Integrated Intermediary Care (CIIC) short-term stay at a respite care facility for older persons.

Type of staff	Number of staff	Function and services
Auxiliary nurses^a^	2	CIIC facility duty; coordination (arranging and scheduling screening and training), technical training of family caregiver for specific care needs, functional training to prevent a need for care
Nurse assistants	2	CIIC facility duty, care of older adults staying at the CIIC (meals, laundry, beds, and personal care), providing technical training for the family caregiver to provide specific care needs
Volunteers	30	Helping with the training of caregivers, social marketing of the CIIC to the community, psychosocial support, assistance with functional training, assisting CIIC staff

^a^These nurses are employed by this research project and are not seconded from the health system.

### Respite Care Service for Families Having Difficulties With Caregiving

In this project, intermediary care is defined as formal care that is neither day care nor a long-term residential service. It is a short-term residential service for older persons whose caregiver is temporarily unable to sustain care at home.

#### Registration

All the families assessed to be eligible for respite care services will be invited to register for the CIIC center’s temporary respite care services, which is not part of the primary health care center of the subdistrict. The services will be provided by newly established CIIC centers staffed with professional staff and volunteers who will provide assistance to family caregivers whenever they find it challenging to sustain caregiving of an older family member. The maximum length of stay is 10 days during the 6-month intervention. Our previous survey study found that 6.5% of older persons had decreased capabilities for ADL that required caregiver assistance [11]. Therefore, the estimated number of persons needing respite care is 130 people in a year; 50% (65/130) may be without a caregiver. These individuals will be provided with residential care; therefore, our calculations show that 5 beds would be sufficient for 10 persons at 2 weeks per year. This might not be enough beds during certain periods of the year. Therefore, a booking service will be provided.

#### Staff

CIIC centers will have 2 auxiliary nurses, 2 nurse assistants, and 30 caregiving volunteers. Auxiliary nurses in Thailand have a bachelor’s degree in public health, underwent an internship at primary health care centers, and received accredited advanced training in caregiving that certifies that they have achieved minimum professional standards and technical quality. Volunteers who received caregiving training will assist the auxiliary nurses.

#### Applicants

The CIIC temporary respite care service will be provided to applicants with full-time, unpaid family caregivers caring for dependent individuals who are ≥60 years old and meet the eligibility as assessed using the ADL and CBI, as described in the following sections.

### ADL Criteria

In order to be eligible to be admitted to the CIIC facility, the family caregiver must be personally providing the care recipient with assistance for at least 2 of the following 6 ADL. The first is bathing: The family caregiver is assisting the older adult with bathing, including help with washing, shampooing, getting in or out of the tub or shower, brushing teeth, and other aspects of personal grooming (Bathing ADL score 0). The second is dressing: The family caregiver is assisting the older adult with dressing, including helping the individual put on or take off clothing and footwear (Dressing ADL score 0 or 1). The third is toileting: The family caregiver is helping the older adult get on or off the toilet, commode, or bedpan as well as clean themselves, or the individual is incontinent (Toileting ADL score 0 or 1). The fourth is transferring: The family caregiver is helping the older adult get to and from a bed or chair (Transfer ADL score 0 or 1). The fifth ADL is walking or mobility: The family caregiver is helping the older adult move from one stationary point to another by removing obstacles, opening doors, and assisting with canes, wheelchairs, or other assistive devices (Mobility ADL score 0 or 1). The sixth is eating or feeding: The family caregiver is helping the older adult who has difficulty chewing or swallowing without assistance or needs partial or total help with eating (Feeding ADL 0 or 1).

### CBI Criteria

On the assessment of caregiver burden, the following 2 eligibility criteria are required to utilize the respite care service: time dependency items score >17 and physical health items score >14.

If there is only one person in the family acting as a caregiver for the older adult with the qualifying ADL criteria and the caregiver has to leave the house to travel, the older adult would qualify for admission to the CIIC based on the available capacity of the facility on the appointment days.

These criteria can be revised by stakeholders depending on the estimates of the population to be served, budget of the municipality, and capacity of the facility.

### Resources

To accommodate short-term stays, the CIIC intervention requires buildings and care facilities. Researchers have discussed this with the administrative officers of the study site subdistrict and obtained commitments to let the CIIC center use public buildings and infrastructure with beds, bathrooms, kitchens, washrooms, air conditioners, televisions, refrigerators, and outdoor facilities such as gardens, walkways, and sports facilities for the 24/7 care of 5-10 older adults.

The CIIC facility will employ dedicated full-time auxiliary nurses and other staff for this research. The CIIC service will not be delivered by existing primary health care nurses. Therefore, the busy routine of the primary health care facility will not affect the CIIC service or be disrupted. Access to health care in case of medical care needs of the participants will be secured by establishing a link between the CIIC and primary health care center before starting the study. In addition, for smooth linkage, the researcher will link to local health facilities that will be coordinated by the local municipal authority before starting the study.

The existing universal health coverage scheme covers health services 100% free of charge. The primary health care services are accessible by any Thai citizen. There is a referral system to the secondary district hospital, and an ambulance service is also available 24 hours a day.

### Outcome Measurements

Validated instruments commonly used in aging and LTC research were carefully selected in order to objectively assess the impact of the CIIC intervention. Most of the instruments exist in the Thai language and have been validated in previous studies and programs in Thailand. In addition, the research team will conduct pilot testing of the instruments for the target population and ensure the reliability of all the instruments in the study setting and context.

#### Primary Outcome

The primary outcome is the burden of family caregivers measured at a 6-month follow-up appointment. The CBI [14,15] will be applied to measure the family caregiver’s burden at baseline (month 0) and then again at month 6. CBI is an internationally well-validated measurement tool with a 24-item, 5-point Likert scale with the following 5 dimensions: time dependence burden (5 items), developmental burden (5 items), physical burden (4 items), social burden (5 items), and emotional burden (5 items).

The total scores will be summed, with total scores >36 indicating a risk of “burning out” whereas total scores >24 are considered indicative of “a necessity to seek some form of respite care.”

#### Secondary Outcomes

Secondary outcomes consist of biopsychosocial indicators including ADL, depression, and QOL of older people, which will be compared between intervention and control groups.

Functional ability will be assessed by applying the Barthel Index for ADL assessment [16], which measures the level of ability for 10 basic items (bowel movement, incontinence, grooming, toileting, feeding, transfer, mobility, dressing, stairs, bathing). Total possible scores range from 0 to 20, with lower scores indicating increased disability.

Depression will be screened by applying the Geriatric Depression Scale (GDS), which is internationally commonly used, validated, and regularly used in Thailand [17].

Health-related QOL will be measured with the EQ-5D-5L questionnaire [18] using the EQ visual analogue scale (EQ VAS) [18,19]. A validated Thai version of the instrument is already available and will be used after piloting. The EQ-5D-5L measures 5 dimensions with 5 levels: mobility, self-care, usual activities, pain/discomfort, and anxiety. The EQ VAS is a measure of overall self-rated health status with a vertical VAS where the end points are labeled “Best imaginable health state” and “Worst imaginable health state.”

Cost for the intervention will be calculated in terms of intervention cost, family cost, and community cost. The details of the intervention cost will inform the estimated cost when expanding the area covered by the CIIC services.

The first possible outcome of the implementation of the CIIC intervention is promoting the capacity of the family caregivers through technical training, early detection of dependency problems, and respite care when the caregiver is overburdened in addition to linking families and primary health care services. The intervention components such as the (1) community respite home and (2) technical capacity building of the caregiver are directly targeting the reduction of caregiver burden.

Therefore, we set caregiver burden as the primary outcome of the study. ADL is also an important outcome of the component. Although we set these as the primary and secondary outcomes, respectively, in terms of the research methodology, they are both important outcomes of the study.

Selecting an outcome that is directly related to most of the intervention practically, we chose caregiver burden as the primary outcome. In a country with an FLTC system as its backbone, this outcome is more practical.

### Sample Size

A sample of 4000 community-residing older adults will be recruited, with 2000 adults in each arm of the study (control and intervention), which our calculations show should suffice for the power required by the study. Including 1500 participants in each arm should be ample to detect the effect size difference of 0.5 unit with an SD of 4 between the 2 arms. STATA version 11SE (Stata Corporation, College Station, TX) was utilized for sample and power estimations. The precision levels applied are a *P* value of .05 with a 95% confidence interval. The sample size is inflated (1) for a design effect of 1.2 for applying a cluster randomized design and (2) to compensate for potential nonresponses and drop-outs of up to 20% during recruitment and the study. After inflation, a total sample size of 4000 is estimated to be required for the study. Moreover, the sample size will be sufficient for the estimation of key parameters such as mean CBI, ADL, and health-related QOL indicators in the study population.

There will be 6 clusters in each arm. The cluster size is in the range of 300-350. Within each cluster, individual participants meeting eligibility criteria will be recruited consecutively. Informed consent will be requested from a representative of each cluster before randomization and from each older participant and a family caregiver after randomization.

### Randomization

#### Sequence Generation

Cluster randomization will be used to prevent contamination between the intervention subdistrict and control subdistrict [20]. It is not possible to randomize the villages from different administrative areas or municipalities and directly assign them to an intervention and control arm. Internal randomization will be practiced to randomly recruit 6 eligible clusters within the intervention arm municipality, which has 10-15 villages; likewise, the same will be done in the control arm [20].

#### Random Allocation

A random number will be generated to recruit villages randomly within each arm. A statistician blinded to the study will generate random numbers for each arm of the study. Control arm and intervention arm villages are geographically distant and administratively exclusive, yet they are in the same province and similar demographically, socially, and culturally to evaluate social interventions.

#### Blinding

Double blinding is not possible. Participants are blind to the allocation. Participants and research assistants performing the assessment cannot know the random allocation to a cluster before the study begins. Bias and contamination are controlled.

### Implementation

**Figure 2:**
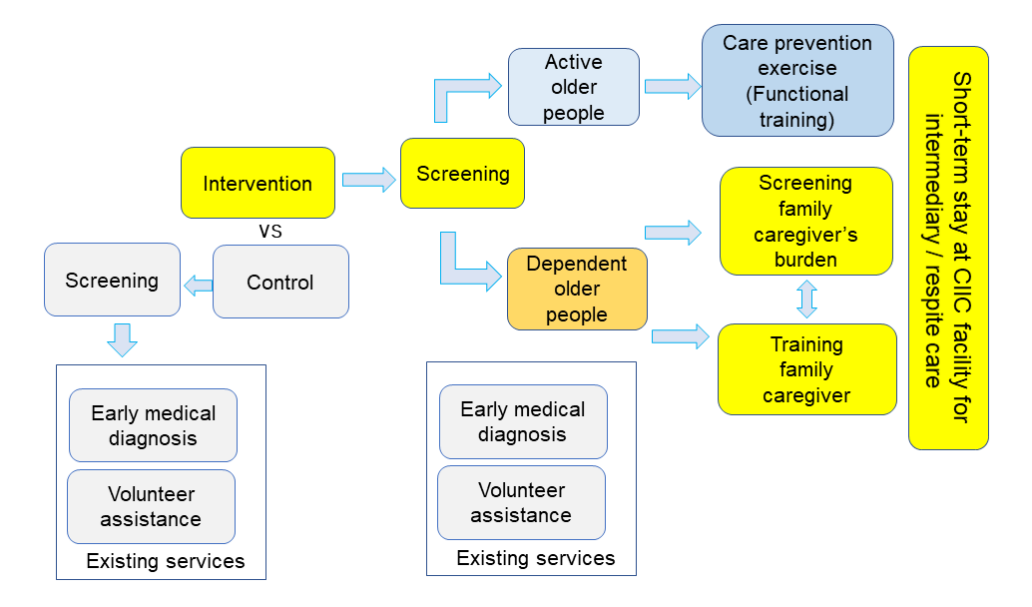
Activities and services in the intervention and control arms of the Community-Integrated Intermediary Care (CIIC) trial, Thailand.

**Figure 3:**
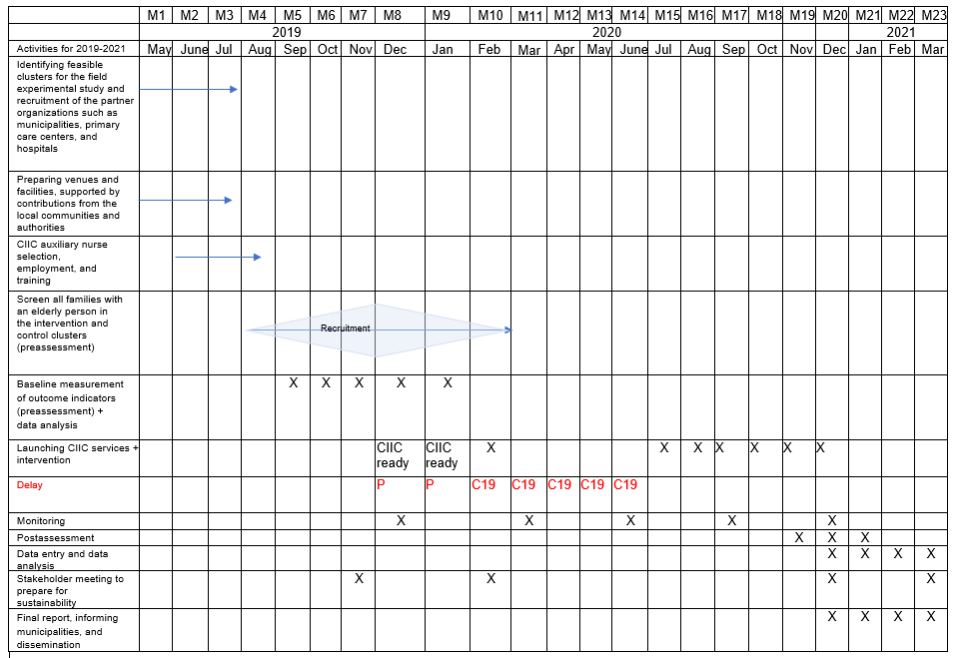
Timeline for the Community-Integrated Intermediary Care (CIIC) project, Thailand. C19: COVID-19 pandemic; P: PM 2.5 air pollution.

The details of the intervention are described in the Methods section (Figure 2). The overall study period is 2 years, which will allow a net intervention period of 6 months, with the remainder of the time spent on preparation for the study such as establishing the facility, recruiting human resources for the service and research implementation, and assessment and evaluation before and after the intervention (Figure 3).

#### Data Collection and Follow-Up

Interviewer-administered, structured questionnaires will be used to collect the data. Follow-up will be comprised of (1) serial measurement of research outcomes, (2) monitoring the implementation for quality control, and (3) monitoring of serious adverse events related to the trial. Serial measurement of research outcomes will be applied, ensuring 6 months of follow-up for each participant. Baseline characteristics will be measured in both intervention and control arms before launching the CIIC service at the outset of the study. Postintervention, primary and secondary outcome measures will be measured again after 6 months of follow-up. There is minimal risk of drop-out from the study as the participants are long-term residents of the subdistricts.

#### Safety and Privacy Considerations

Confidentiality of data from the screening and intervention will be maintained by storing all paper-based patient material in locked cabinets accessible only to authorized personnel. Names or identity numbers of the patients will be excluded from the database. The databases are password protected and accessible only to authorized personnel and researchers. Participants will be informed that they are free to decide to leave the study without any impact on their privilege under the national health insurance.

### Ethics and Trial Registration

The research project has been approved by the World Health Organization (WHO) Ethical Review Committee (WHO/ERC ID; ERC.0003064) and Boromrajonani College of Nursing, Lampang Thailand Ethics Review Committee (E2562/005). It has been registered in the Thailand Clinical Trial Registry, with trial registration number TCTR20190412004.

### Statistical Methods

The analysis will follow the intention-to-treat analysis approach. Outcomes will be compared between intervention and control clusters after 6 months of intervention.

To assess the effectiveness of the intervention, the primary outcome, CBI, will be compared between intervention and control arms after 6 months of intervention. Depending on the nature of the variables, *t* tests, chi-square tests of cluster summary measures, or multivariate analyses will be applied. Data distribution and homogeneity will be checked and ensure the assumption of statistical tests. Cluster-level summary statistics will be compared. A random effects model will be applied to control possible confounders in individual and cluster levels.

The intervention in the CIIC trial is a complex intervention with several components of prevention, training, and service. It is basically empowerment of the community. Therefore, a village as a random effect is due to population and social economic status, and participation of the community is considered as source of variation.

To evaluate the impact of the intervention on the secondary outcomes (ADL for dependency, GDS for depression, and EQ-5D-5L for QOL), the analyses will compare the 2 arms by applying a multivariate analysis of variance model.

Afterwards, the cost of the intervention will be analyzed in terms of both the primary outcome and secondary outcomes. Statistical significance is defined as *P*<.05 with 95% confidence intervals.

## Results

The CIIC facility has been established and equipped to provide care services to older persons who requested and were eligible to receive the service. Community-based care prevention programs have been launched in the intervention clusters. Nurses from the CIIC visited the houses of older persons upon the family caregiver’s request for care planning advice. Family caregivers received training and assistance. However, the COVID-19 pandemic delayed the interventions (Figure 3).

Care prevention exercises have been launched in the communities of the 6 intervention clusters. The adoption rate was 100%. Community stakeholders, municipality authorities, and the Ministry of Public Health of Thailand actively collaborated with the research team. The community empowerment approach consisted of training volunteers to be trainers of the exercise groups, providing group exercise DVDs and televisions to use in the group exercise to each cluster, providing home exercise DVDs and posters for maintaining exercise at home, a calendar to record exercise frequency, and a line group for communication among the participants. Community-based, functional-training, group exercise activities were launched in February 2020. The research project provided DVDs free of charge to the intervention arm participants. During the suspension of the community-based activities due to the COVID-19 pandemic, care prevention exercises were sustained by applying home exercise.

The COVID-19 pandemic and declaration of emergency postponed the intervention program until June 2020. Afterward, the program was resumed. The study was implemented until December 2020. Since then, the CIIC trial is undergoing evaluation. The evaluation will be completed by the end of March 2021. The timeline for study implementation could be extended by the local ethical committee and WHO Ethical Review Committee.

## Discussion

### Principal Findings

Health and social care that is accessible in a universally covered scheme is an expectation of all second wave-aging countries like Thailand. They need an aging care model that can enhance FLTC and link to primary health care services, while being integrated into the community and local government in each municipality. CIIC is expected to provide a potential model that can link families and communities to local formal services and funding. In addition, it will promote active aging through a community-based care prevention exercise program for older persons.

The CIIC facility and services will introduce formal intermediary care services to the Thai community whereby informal care will be strengthened through helping families to improve their capacity for the care of older people [7]. Locating CIIC services in a neighborhood environment is designed to raise local attention and people’s awareness, familiarity, and trust within the social network of the local community [21]. This is a strategy to realize the integration of older people into community care services.

**Figure 4:**
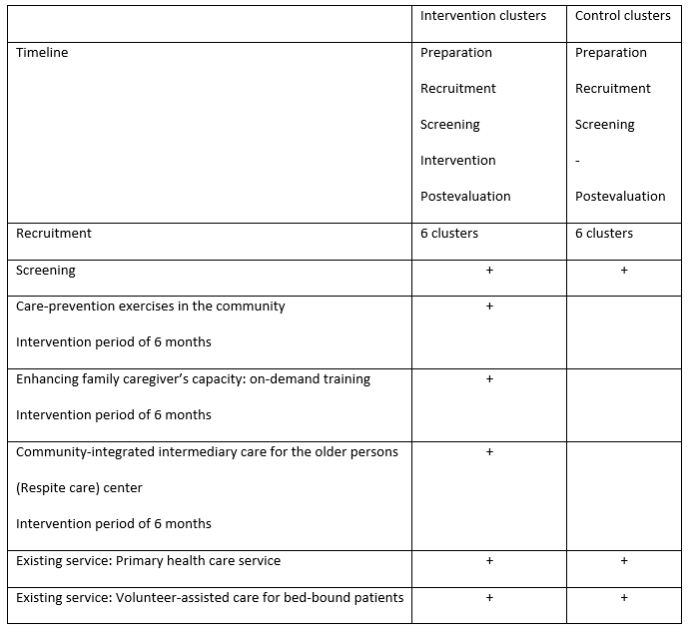
Components of the intervention in the 2 arms of the Community-Integrated Intermediary Care (CIIC) trial.

Furthermore, we introduced 3 steps as part of a prevention strategy in the CIIC services (Figure 4). The first step is a community LTC prevention exercise program co-created to function as community empowerment. Participation in such community exercises can promote autonomy and active aging, with the potential outcome that will prevent dependency and need for LTC [22]. Second, when there is little care need, the older person can enjoy staying at home with their families, thereby improving their chances of “aging in place” [4]. When family caregivers face burnout, older persons will be able to access a formal care service in the community. Overall, families will be assisted through training, direct help, and a respite service provided by the CIIC [9]. Again, the hope is that it can prevent unnecessary LTC costs. Third, when older people remain active and healthy, health care needs may be reduced, thereby avoiding unnecessary hospital admission and saving health care costs. Screening of ADL and for common noncommunicable diseases will identify unmet needs earlier and prevent age-related morbidity and disability. Therefore, in the long-term, the interaction between the CIIC and primary health care services could be harmonized, leading to better integration and could be a step to realizing universal access to long-term and health care in Thailand.

#### Dissemination of Results and Publication Plan

A lay summary of the results will be communicated back to the communities via community radio and relevant social media outlets. The results will be reported back to the WHO Kobe Centre, published in international peer-reviewed journals, and relayed to the Ministry of Health of Thailand, local authorities, and development partners in aging and LTC services in the Asia-Pacific region.

#### Future Implications

With the CIIC model, the strategy for sustainability could be realized through mobilizing community participation and assets towards the shared value of active aging and sustainable social care for the older person. Therefore, we expect that the model of intermediary care in this study will yield a sustainable, affordable, and universally accessible model that could enhance FLTC in Thailand and other similar rapidly aging countries in Asia.

#### Challenges and Limitations

Cluster randomization may increase the risk of imbalance in the 2 arms at baseline. This approach of randomization we practiced in CIIC may have limitations but would minimize the residual confounders caused by differences in characteristics of the municipalities. However, this is the best pragmatic approach for the current trial. The results of randomization should be checked carefully at baseline by comparing the aggregate-level summary.

We do not have data about the desirability of temporary, short residential stays at the CIIC facility for older adults and their families. We may have underestimated the demand and therefore, also the required resources. Researchers have consulted with local governments about the possibility of tapping into reserve capacity and resources. Social distancing measures in the COVID-19 pandemic delayed the activities, but we adhered to those measures to ensure the safety of participants.

#### Conclusion

With a rapidly aging population, sustainable health and social care provided to older people in their community are urgently required. We see this as a potential step to the realization of universal health coverage and well-established primary health care services that are inclusive of aging populations in Thailand. The evidence and lessons learnt in this study are expected to be beneficial to other similar countries in Asia.

## Abbreviations

ADL: activities of daily living
ASEAN: Association of Southeast Asian Nations
CBI: Caregiver Burden Inventory
CIIC: Community integrated intermediary care
EQ-5D-5L: EuroQol 5-dimensions 5-levels
FLTC: family-based, long-term care
GDS: Geriatric Depression Scale
LTC: long-term care
QOL: quality of life
VAS: visual analogue scale
WHO: World Health Organization
